# Immune Factors Drive Expression of SARS-CoV-2 Receptor Genes Amid Sexual Disparity

**DOI:** 10.3390/v15030657

**Published:** 2023-02-28

**Authors:** Ashutosh Vashisht, Pankaj Ahluwalia, Ashis K. Mondal, Harmanpreet Singh, Nikhil S. Sahajpal, Sadanand Fulzele, Vamsi Kota, Gagandeep K. Gahlay, Ravindra Kolhe

**Affiliations:** 1 Department of Pathology, Medical College of Georgia, Augusta University, Augusta, GA 30912, USA; 2 Department of Molecular Biology and Biochemistry, Guru Nanak Dev University, Amritsar 143005, India; 3 Greenwood Genetic Center, Greenwood, SC 29646, USA; 4 Department of Medicine, Medical College of Georgia, Augusta University, Augusta, GA 30912, USA

**Keywords:** COVID-19, SARS-CoV-2, immune response, coronavirus

## Abstract

The emergence of COVID-19 has led to significant morbidity and mortality, with around seven million deaths worldwide as of February 2023. There are several risk factors such as age and sex that are associated with the development of severe symptoms due to COVID-19. There have been limited studies that have explored the role of sex differences in SARS-CoV-2 infection. As a result, there is an urgent need to identify molecular features associated with sex and COVID-19 pathogenesis to develop more effective interventions to combat the ongoing pandemic. To address this gap, we explored sex-specific molecular factors in both mouse and human datasets. The host immune targets such as TLR7, IRF7, IRF5, and IL6, which are involved in the immune response against viral infections, and the sex-specific targets such as AR and ESSR were taken to investigate any possible link with the SARS-CoV-2 host receptors ACE2 and TMPRSS2. For the mouse analysis, a single-cell RNA sequencing dataset was used, while bulk RNA-Seq datasets were used to analyze the human clinical data. Additional databases such as the Database of Transcription Start Sites (DBTS), STRING-DB, and the Swiss Regulon Portal were used for further analysis. We identified a 6-gene signature that showed differential expression in males and females. Additionally, this gene signature showed potential prognostic utility by differentiating ICU patients from non-ICU patients due to COVID-19. Our study highlights the importance of assessing sex differences in SARS-CoV-2 infection, which can assist in the optimal treatment and better vaccination strategies.

## 1. Introduction

The emergence of the severe acute respiratory syndrome coronavirus-2 (SARS-CoV-2) pandemic has posed a global health emergency with approximately 649 million people infected and 6.6 million deaths as of 21 December 2022 [1]. As the COVID-19 pandemic continues to evolve, there is a growing need to better understand the risk factors associated with disease severity. Advanced age and comorbidities such as chronic respiratory disease, cardiovascular disease, diabetes, and hypertension have been identified as primary risk factors for severe COVID-19 [2,3,4,5]. Furthermore, several studies have demonstrated sex-related differences in COVID-19 cases and mortality rates, with males exhibiting higher rates of severe and fatal cases [6,7,8,9,10]. Clinical studies on COVID-19 have also established that males are at a greater risk of poor prognostic outcomes and higher mortality rates [10,11,12]. However, the molecular-level sex-specific differences in the host response to SARS-CoV-2 have not been clearly defined yet.

SARS-CoV-2 is a β-coronavirus with positive-sense single-stranded RNA as the genetic material (subgenus: *sarbecovirus*, subfamily: *Orthocoronavirinae*) [11]. The genome sequence analysis of SARS-CoV-2 was found to be 96.2% identical to the bat coronavirus RaTG13, whereas it was 79.6% identical to SARS-CoV [12]. The deep meta-transcriptomic sequencing results showed that the receptor-binding domain (RBD) of the spike (S) glycoprotein of SARS-CoV-2 was one amino acid longer than that of SARS-CoV [13]. An analysis of bronchoalveolar lavage fluid (BALF) from a COVID-19 patient along with infectivity studies in HeLa cells concluded that just like SARS-CoV, SARS-CoV-2 also uses angiotensin-converting enzyme 2 (ACE2) as the cellular entry receptor, and hence is capable of direct human transmission [12,14]. Transmembrane serine protease 2 (TMPRSS2) forms a receptor-protease complex by associating with ACE2 and allows for the successful entry of the virion into the cell [15]. SARS-CoV-2 RNA has been reliably detected in bronchoalveolar lavage fluid, nasopharyngeal swabs, sputum, blood, and stool samples, although with differential sensitivity [16].

The severity of COVID-19 disease is thought to be linked to virus-induced damage to cells and the ability of the virus to evade the host immune system [17]. In COVID-19 patients, the immune system can induce a lethal inflammatory condition known as cytokine release syndrome (CRS) [18]. This phenomenon involves an extreme inflammatory response, where large amounts of inflammatory cytokines are rapidly secreted in response to infective stimuli. Patients with severe COVID-19 exhibit higher levels of pro-inflammatory cytokines (such as IL-6 and IL-8) in their bronchoalveolar lavage fluid as well as increased expression of inflammatory chemokines (such as CCL2) in macrophages compared to those with mild COVID-19 [19]. The unconstrained cytokine storm is particularly severe in patients requiring admission to an intensive care unit (ICU) [20]. However, defining the clinical criteria for CRS remains challenging, and the mechanisms responsible for the unchecked release of inflammatory factors are still unclear.

Several studies suggest that viruses have developed strategies to counteract the host defense system to ensure their survival and propagation. The first viral protein that was found to interfere with the host immune system by targeting TLRs was A46R of the vaccinia virus (VACV) [21]. The A46R protein directly regulated the TIR-domain-containing adaptor proteins. Additionally, studies have shown that pathogen recognition receptors play an important role in viral entry to the cells. In a study conducted on CXCR4 (a chemokine receptor acting as an HIV co-receptor), it was found that CXCR4 was a component of the TLR oriented receptor complex active in LPS recognition [22]. In a similar study, the oral/systemic pathogen *P. gingivalis,* which causes periodontal/systemic infections, evades TLR-mediated immunity by binding to CXCR4, which induces PKA signaling. This, in turn, inhibits TLR2-mediated proinflammatory and antimicrobial responses, and thus resists its clearance from the body [23,24]. In another study on Epstein–Barr virus (EBV), it was suggested that the virus modulates the TLR7 pathway to regulate IRF-5, which has antiviral activity, to minimize the immune response [25]. EBV was found to induce a new negative regulatory IRF-5 splice variant, V12, which had no activation domain but was able to code for a DNA binding domain. This led to the inactivation of the immune response in the presence of EBV.

The effect of sex steroid hormones has resulted in sex-specific disease outcomes in many diseases [26,27]. While testosterone suppresses innate immune response [28], the presence of estrogen (ESR) at higher concentrations is immune-suppressive and at lower concentrations, is immunomodulatory [29]. Estrogen signaling can also promote adaptive T-cell response [30] and can limit influenza infection by modulating genes that regulate the cells’ metabolic function [31]. Additionally, androgen receptors (AR) are involved in the severe outcome of viral infections such as COVID-19 and hepatitis B virus (HBV) [32,33]. A lot of information is known about the immune response generated by SARS-CoV-2 infection, however, key issues related to SARS-CoV-2 pathogenesis and its sex-specific outcome in humans are still to be resolved.

In this manuscript, we have offered a perspective on the possible interaction of SARS-CoV-2 with the host immune system and discussed scenarios through which these interactions have resulted in increased severity of the infection and its association with sex-specific outcomes. While previous analysis of the host response to SARS-CoV-2 highlighted a role for TLRs, IFNs, IL6, and other immune factors, the cell-specific studies elucidating their association have not been explored yet.

Single-cell RNA-sequencing (scRNA-Seq) is reshaping our potential to comprehensively analyze numerous types of cells during healthy and infectious states. The global single-cell sequencing databases such as single-cell portals have enhanced our understanding of infections during such pandemics [34,35]. In this paper, we have used such datasets (all from non-SARS-CoV-2 infected samples) to analyze mouse cells that have been assigned as SARS-CoV-2 targets and investigated the co-expression of the above-mentioned host immune factors. We focused our analysis on airway epithelial cells, which were found to be the primary target of SARS-CoV-2 spread in the lungs. After analyzing the single-cell RNA databases, we found that the expression of TLR7, IRFs, IL6, estrogen receptors, and the androgen receptors were associated with SARS-CoV-2 cell receptors. Additionally, the genome-related analysis showed that the estrogen receptor subtype was under the immediate control of the ACE2 promoter and androgen receptor (AR) under the IRF5 promoter region. To further understand the variations in the gene expression level, we utilized two independent external datasets. The comparison of a 6-gene signature (ACE2, TMPRSS2, AR, TLR7, IL6, and IRF5) was made to explore the sex-based differences associated with COVID-19. These findings point to a potential sexual disparity and a strong influence of underlying immunological components in determining the susceptibility to COVID-19 associated illness.

## 2. Results

Human Cell Atlas datasets have shown that *ACE2* and *TMPRSS2* are expressed in the nasal, lung, and gut epithelial cells [36]. The highest expression of both these genes has been found in nasal goblet cells and multi-ciliated cells, which showed that these cells have a significant role in reservoiring viral load. Furthermore, in the distal lung, co-expression has been found in alveolar type-2 (AT2) cells [37,38]. However, these studies have not included the data related to immune factors and thus lack the results that could explain the possible role of immune response in regulating the expression of SARS-CoV-2 receptor genes. To address this limitation, we focused on immune genes that play a role in the initial response following the detection of viral RNA such as Toll-like receptors (TLRs), interferon regulators, and cytokines as well as SARS-CoV-2 host receptor proteins (Table 1). We further examined whether immune responses to SARS-CoV-2 vary between males and females and whether these differences are associated with the observed disparities in the disease course of COVID-19. To accomplish this, we analyzed the AR and ESSR genes in our datasets. Only single-cell RNA studies were chosen in this study, which included the expression data of the desired candidates.

### 2.1. SARS-CoV-2 Receptor Genes and Host Immune Factors Co-Expression in Airway Epithelium

**Figure 1:**
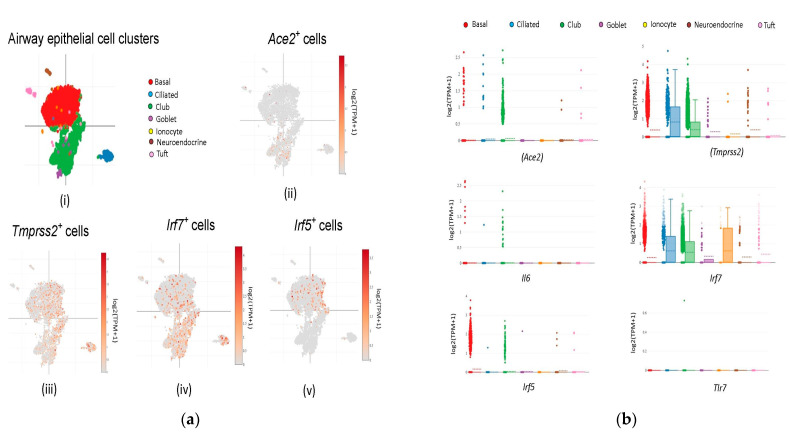
(**a**) Single-cell sequencing profile of the mouse airway lung epithelial cells. (**i**) t-SNE color-coded view of all the 7193 airway epithelial cells of the mouse. Differential expression of (**ii**) *Ace2* (**iii**) *Tmprss2* (**iv**) *Irf7,* and (**v**) *Irf5* genes in the epithelial cells. (**b**) Expression profile of targets along with the SARS-CoV-2 receptor genes in the mouse airway lung epithelial cells. Box plots showing the differential expression of *Ace2*, *Tmprss2*, *Il6*, *Irf7*, *Irf5*, and *Tlr7* in various epithelial cells.

To investigate whether candidate immune factors are directly associated with the SARS-CoV-2 receptor genes and if there is any co-localization and expression pattern present among these, we performed a set of bioinformatics analysis. String analysis of the host genes including immune factors-*TLR7*, *IRF5*, *IRF7*, and *IL6* along with the SARS-CoV-2 receptor genes (*ACE2*, *TMPRSS2*) was performed to analyze any functional interaction between the target proteins. The string analysis result showed that the immune factors were actively associated with ACE2 and TMPRSS2 through IL6 (Figure S1). The STRING parameters showed the following: the number of nodes, 6; number of edges, 8; average node degree, 2.67; average local clustering coefficient, 0.75: expected number of edges, 1; PPI enrichment *p*-value, 3.54 × 10^−6^ (Table S1). Furthermore, to determine whether the candidate genes were co-localized with the SARS-CoV-2 receptor genes, we performed a targeted analysis of *Ace2*, *Tmprss2*, *Tlr7*, *Il6*, *Irf5*, and *Irf7* gene expression in the mouse lung airway epithelium. We examined these genes in a curated dataset of 7193 airway epithelial cells for gene expression from the tracheal portion of the lung. These cells were investigated broadly in the regions of the basal, ciliated, club, goblet, ionocyte, neuroendocrine, and tuft cells (Figure 1a). All candidate genes were expressed in the airway with the highest levels observed for *Tmprss2*, and *Irf7* and the lowest level observed for *Il6* and *Tlr7* (Figure 1b). Club cells showed the highest expression of candidate proteins. *Irf7* and *Tmprss2* showed expression in almost all of the cells, whereas *Tlr7* expression was confined to club cells only. *Ace2*, *Il6*, and *Irf5* were variably expressed in the basal, ciliated, and club cells. Together, the results of the STRING analysis and curated scRNA sequencing showed that immune factors could regulate the expression of Ace2 and Tmprss2 as they tend to share co-expression and localization patterns across the airway epithelial cells.

### 2.2. X-Chromosome-Related Genes May Influence the Severe Outcome of SARS-CoV-2 in Males via IRF Expression

**Figure 2:**
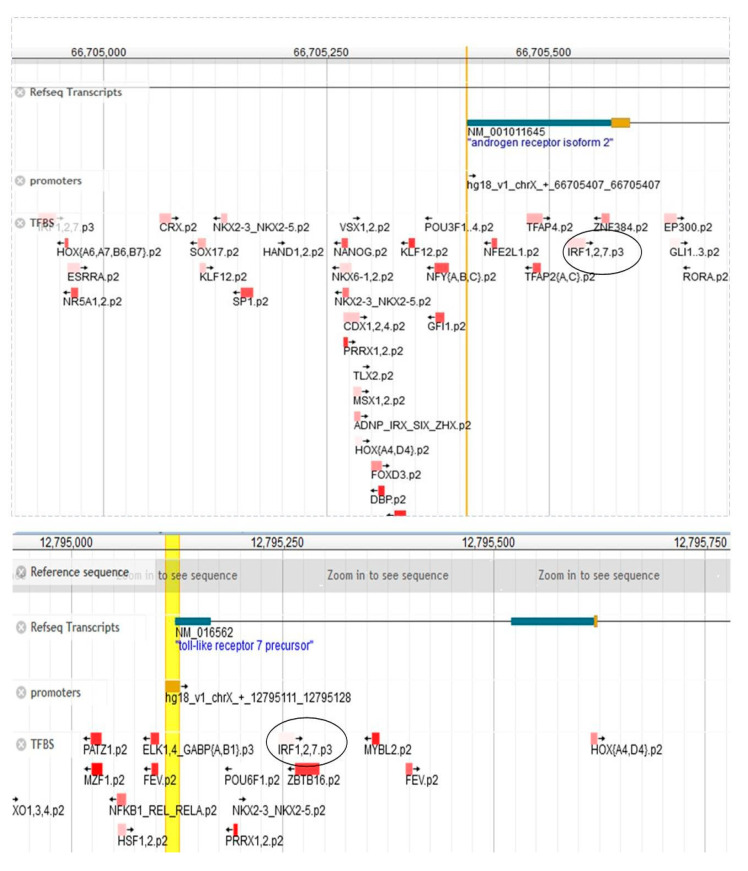
Swiss regulon analysis data showing IRFs under the AR and TLR7 promoter region. The promoter region analysis showed that AR and TLR promoters have been directly associated with IRFs. This can be exploited by SARS-CoV-2 to initiate the cytokine storm, which could lead to tissue injury in COVID-19 patients.

We next sought to understand how the expression of X-chromosome-related genes may relate to covariates that have been associated with disease severity in a particular gender during COVID-19. Among the shortlisted gene candidates, AR, ACE2, and TLR7 were found to be present on the X chromosome. Additionally, the co-localization and expression analysis described above identified an association between the IFN signaling pathway and the SARS-CoV-2 receptor genes. This prompted us to investigate whether IFNs may play an active role in regulating the ACE2 expression levels and thus potentially allow for a positive host response for viral entry. We performed the string analysis of genes including AR, ACE2, TMPRSS2, and the above-mentioned immune factors. It was observed that the SARS-CoV-2 receptor genes were associated with IRF5 via IL6 and AR (Figure S2). The STRING parameters showed the following: the number of nodes, 6; number of edges, 7; average node degree, 2.33; average local clustering coefficient, 0.361: expected number of edges, 1; PPI enrichment *p*-value, 0.00022 (Table S2). Furthermore, the human promoter region analysis showed that the AR promoter at the chrX:66704897-66705917 and TLR7 promoter at position chrX:12795111-12795128 might regulate the expression of IRFs (IRF1, IRF2, IRF7) at chrX:66705521-66705540 (+strand) and chrX:12795245..12795264 (+strand), respectively (Figure 2). In a sex-based meta-analysis study conducted on COVID-19 patients by Huang et al. [3], the results showed that AR could regulate the expression of ACE2 and TMPRSS2, which can dictate the possible role of sex hormones in disease outcome. However, our analysis tried to decipher the hidden link of immune factors in this cascade of reaction, which may influence the expression of SARS-CoV-2 receptor genes. Based on these analyses, it can be hypothesized that SARS-CoV-2 can use the upregulation of IRF7 and IL6 as a double-edged sword by increasing the pro-inflammation amid tissue injury and by increasing the expression of ACE2 and TMPRSS2 for its entry to infect the cells. This further strengthened our hypothesis that X-related genes can be influenced to accelerate the SARS-CoV-2 infection in the cells.

### 2.3. Estrogen Expression under the Influence of ACE2 in Females Can Be Protective during SARS-CoV-2 Infection

**Figure 3:**
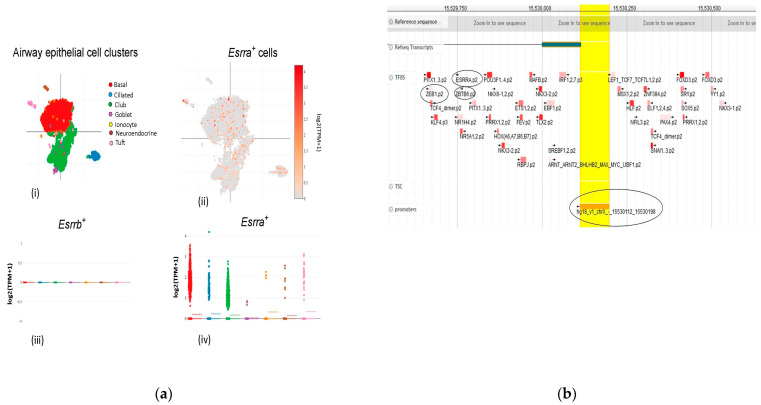
(**a**) Single cell-RNA sequencing co-localization and the expression profile of estrogen receptors present mouse airway lung epithelial cells along with the SARS-CoV-2 receptor genes. (**i**) t-SNE color-coded view of all the 7193 airway epithelial cells of a mouse along with (**ii**) the differential expression of ESRRRA and (**iii**,**iv**) the expression profiles of *ESRRA* and *ESRRB* expressing epithelium cells. (**b**) Swiss regulon analysis data showing the estrogen receptor alpha and zinc finger protein ACE2 promoter region. The promoter region analysis showed that ACE2 can regulate the activity of ESRRA and zinc finger proteins. This can be used by the host immune system to counteract SARS-CoV-2 in females.

Various studies have mentioned the protective nature of estrogen in females during viral infections, however, the underlined mechanism has yet to be explored. In a quest to investigate the role of estrogen during SARS-CoV-2, we first analyzed the sRNA sequencing data of the same mouse airway epithelial cells to observe the co-localization and expression of the female sex hormone receptors estrogen-related receptor alpha (ESRRA) and estrogen-related receptor beta (ESRRB) among the SARS-CoV-2 receptor genes. The results showed that ESSRA was expressed in all of the cells (higher in the basal cells), but ESSRB was not detected (Figure 3a). It is important to mention here that the basal cells also showed a higher expression of ACE2. This prompted us to perform further investigation, which was based on human promoter region analysis. The ChiP sequence analysis showed that ESSRA at chrX:15529756.15529769 (-strand) was under the regulation of ACE2 promoter region chrX:15530112..15530198 (-strand) (Figure 3b). Additionally, the zinc finger protein at chrX:15529667..15529678 (-strand) was found to be downstream of ESSRA during this analysis, and zinc fingers have been associated with antiviral properties [46]. Based on these results, it can be hypothesized that during the viral infection, ACE2 overexpression upregulates ESSRA, which in turn accelerates the expression of antiviral, zinc finger proteins. This cascade could be a major reason why females have less severe outcomes during SARS-CoV-2 compared to males.

### 2.4. Clinical Implication of Gene Signature in Human Infection

**Figure 4:**
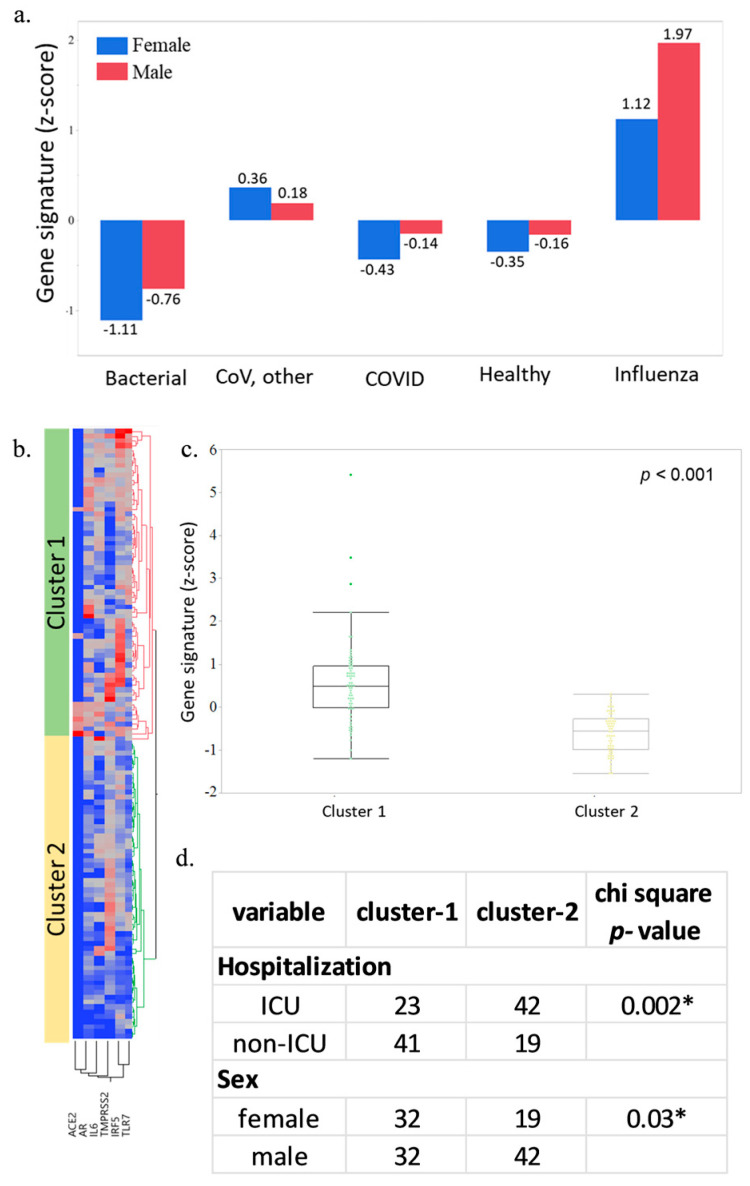
Differential gene expression analysis based on a 6-gene signature. (**a**) Median expression of a 6-gene signature indicating the sexual disparity between males and females. (**b**) Hierarchical clustering based on a 6-gene signature with two distinct clusters (Cluster 1 and Cluster 2). (**c**) Distribution of z-score in different clusters. (**d**) Differential expression based on hospitalization status and sex. The comparison in cluster analysis was made using the Wilcoxon rank-sum test, while the Chi-square test was used for a comparison of clinical variables. * represents the *p* < 0.001 significance.

We further explored the clinical implications of our gene signature in two independent datasets. The expression of the 6-gene signature was found to be higher in males compared to females (Figure 4a). In another dataset, hierarchical clustering identified two clusters with significant variation in gene expression. Cluster 1 had a mean of 0.578 and cluster 2 had −0.611 (z-score) (Figure 4b,c). Chi-square analysis identified an association of higher expression of the 6-gene signature with non-ICU patients, along with a significant sexual disparity based on gender.

## 3. Discussion

Understanding the potential gene interactions across different cells is important to interpret viral infection and the immune response activated against it. Several studies have been performed to understand the tissue-level expression of SARS-CoV-2 receptors in the human epithelia of the lung [47,48,49]. However, unlike ACE2 and TMPRSS2, the expression of host immune factors and sex genes related to SARS-CoV-2 remain unknown. Identifying the association of SARS-CoV-2 with host factors is critical for understanding and modulating host defense mechanisms and viral pathogenesis.

In this study, we investigated the target genes that communicated with the SARS-CoV-2 receptor genes in the mouse airway epithelial cells. Our analysis illustrated the crosstalk between various host factors with ACE2 and TMPRSS2 expression. The above-mentioned results describe how the viral proteins have affected the immune response by binding to the immune components before or after the expression of nuclear factors. Through this study, we suggest an alternate hypothesis that the virus does not inhibit the immune response but thrives on it. The host immune response plays a crucial role in the fight against viruses. However, this response can be manipulated by viral genes for survival and propagation. Our analysis found that SARS-CoV-2 can use host immune factors to upregulate ACE2 and TMPRSS2. Our study showed that the mouse airway epithelium enriched for ACE2 and TMPRSS2 had the highest expression of IRFs genes. We extended our investigation through ChiP and promoter region analysis and observed that IRFs could regulate ACE2 expression via AR in males. This could be linked to the higher CFR in males due to SARS-CoV-2. Our analysis is in line with the other studies that showed that the coronavirus has evolved to take advantage of immune response by hampering the IFN signaling pathway for promoting efficient viral entry to the cell [50,51].

In a COVID-19 hypercytokinemia-based study, patients were observed to have upregulation of IL-1, IL-2, IL-4, IL-7, IL-10, IL-12, IL-13, IL-17, GCSF, macrophage colony-stimulating factor (MCSF), IP-10, MCP-1, MIP-1α, hepatocyte growth factor (HGF), etc. [36]. I-sHLH, a type of hemophagocytic lymphohistiocytosis (HLH) is caused by microbial infections (mainly viruses) and is a hyperinflammatory syndrome that leads to multiple organ failure [52]. Crucial features of I-sHLH include high fever, hyperferritinemia, and ARDS (acute respiratory distress syndrome) [53]. ARDS has been the leading cause of death in COVID-19 patients. In a recent study comprising 150 patients of COVID-19 in Wuhan, China, higher levels of ferritin and IL6 were observed in the non-survivors compared to the survivors [54]. It can be hypothesized by analyzing these results and our analysis that IL6 upregulation not only damages the organs of the patients but also helps in the increase of viral load by providing higher expressions of ACE2 and TMPRSS2. All of these targets are exploited by SARS-CoV-2 as a component of a bigger complex for its propagation.

In a mouse-adapted SARS-CoV virus (MA15) study, it was observed that gonadectomized female mice had a gradual weight loss and a higher death rate than the control group. In a different experiment, the treatment of female mice with ICI-182, 780 (Faslodex; an estrogen receptor antagonist) made the group more vulnerable to MA15 infection in contrast to the female mice that were given estrogen agonists [55]. Our analysis also provided the data that ACE2 can enhance estrogen regulation in females during COVID-19 and thus counteract SARS-CoV-2 infection.

Our exploration of gene signatures in human datasets identified significant perturbations associated with sex. Additionally, higher gene expression was associated with non-ICU patients, signifying the classification potential of our gene signature. Recently, several gene signatures that can classify COVID-19 patients have been identified [56,57,58]. These studies, along with our study focusing on sex-based differences, can help in the clinical management of infected patients.

As the present study included the single-cell RNA sequencing data of mice, analyzing these targets in human airway epithelial cells and the careful consideration of appropriately selected gene lists and model cellular systems to understand the pathophysiology of SARS-CoV-2 are highly required. Our results anticipate that transcriptional response to the virus will need to be rigorously characterized in appropriate model systems. Whether the immune factors and X-chromosome-related genes are net protective or detrimental to the host may also depend on the stage of viral infection in a specific host.

## 4. Strengths and Limitations

(i) Our research delves into the sex-specific differences in gene expression during COVID-19 infection in both mice and humans, bridging the gap between basic biology and clinical significance. Our findings have explored the underlying factors that contribute to the varying degrees of COVID-19 susceptibility and severity between the sexes.
(ii) While we utilized two distinct datasets in our analysis, independent analysis in larger datasets, diverse geographic locations, and clinical settings is crucial for the validation of our results. We plan to conduct a clinical validation in-house using our samples.
(iii) It is important to consider various ethno-demographic features such as age, hormonal status (estrogen and testosterone), pregnancy, and chronic conditions in future studies. A well-designed prospective study with appropriate inclusion and exclusion criteria would greatly expand our understanding of the interplay between sex and COVID-19 pathogenesis.
(iv) Our study focused only on a single time point in the post-infection phase of COVID-19 patients. Gene expression is highly dynamic, and the lack of temporal variables has limited our view of the dynamic status of the immune system.

## 5. Materials and Methods

### 5.1. Analysis of Protein Network

The search tool for retrieval of interacting genes (STRING) database, which integrates both known and predicted protein–protein interactions (PPIs), was applied to investigate the co-expression network of the immune markers [59]. The active interaction sources including text mining, experiments, databases, and other co-expression analysis were used by STRING to construct the PPI networks and the outcomes represented as nodes and edges. The nodes correspond to the proteins and the edges represent the interactions. The following steps were taken to conduct the functional enrichment analysis: the multiple protein mode was selected as a total of seven target proteins (ACE2, TMPRSS2, AR, TLR7, IL6, IRF5, and IRF7) taken as input proteins, the interaction score was in the range between 0.400 and 0.700 (medium to high confidence), the maximum number of interactors for the first and second shell was taken as the query protein only, all the active interaction sources such as text mining, experiments, databases, co-expression, neighborhood, gene fusion, and co-occurrence were checked.

### 5.2. Target Localization

Furthermore, to have a comprehensive understanding of immune markers in the different compartments of mouse airway epithelial cells, a single-cell portal was used. Since the number of the cluster-based algorithms relied on gene signatures, we made a concerted effort to find datasets that contained our target gene signatures. Additionally, we looked for the cell type labels and raw counts of the cells. The data from gene expression experiments using Illumina-based single-cell RNA sequencing were obtained. The t-distributed stochastic neighbor embedding (t-SNE) graphs containing the scRNA-Seq expression of 7193 mouse trachea cells which included 3845 basal cells, 425 ciliated cells, 2578 club cells, 65 goblet cells, 26 ionocytes, 96 neuroendocrine cells, and 158 tuft cells were taken for further analysis.

### 5.3. Target Regulation Analysis

To analyze the transcriptional regulation and to extract the precise positional information of the transcriptional start sites of eukaryotic mRNAs, the database of transcription start sites (DBTSS) was used [60]. All of the targets (ACE2, TMPRSS2, AR, TLR7, IL6, IRF5, IRF7, ESSRA, and ESSRB) were individually analyzed to check whether one of these were under the promoter region of the other. Additionally, the Swissregulon portal was used to access the genome-wide annotations of the regulatory sites and motifs [61]. The reference-seq transcripts, transcription factor, and promoters were marked as the available filters for the analysis.

### 5.4. Analysis of Gene Expression Variations in Humans

To evaluate the variation in gene expression and sexual disparity, we explored two datasets, GSE161731 (N = 198 samples) and GSE157103 (N = 126 samples). The datasets were normalized to counts per million for the analysis. Z-score was calculated for each sample based on the standard deviation from the mean for the combined gene signature. Hierarchical clustering of the 6-gene signature was performed and divided patients into high and low clusters. To correlate the gene signature with clinical variables, the chi-square test was used. All statistical analyses were performed using JMP (JMP Statistical Discovery Software (2022) Version 16. SAS Institute Inc., Cary, NC, USA) and R software (R Core Team (2022) R: A Language and Environment for Statistical Computing. R Foundation for Statistical Computing, Vienna, Austria).

## 6. Conclusions

The pandemic caused by COVID-19 highlights the need to understand molecular features associated with its pathogenesis and its association with the clinical and ethno-demographic variables. To understand the pathophysiology of COVID-19, it is essential to correlate the findings from mouse models to better understand the clinical outcomes in humans. In this study, we attempted to explore the sexual differences at the molecular and gene expression levels in both the mice and humans. We identified a 6-gene signature that showed significant variation at the gene expression levels between the males and females along with hospitalization risk groups. The observed variation in gene expression between the males and females can provide insights into the underlying mechanisms that contribute to the varying degrees of COVID-19 susceptibility and severity. The approach can further assist in patient classification, leading to improved clinical management and treatment strategies. Further research is required to investigate the role of other important covariates such as hormones, age, BMI, co-morbidities, vaccination status, and other host factors on SARS-CoV-2 susceptibility and severity. The emergence of new variants and vaccine efficacy may also affect the clinical presentation of sex-specific differences in COVID-19 pathology. A further understanding of the molecular determinants of COVID-19 is essential to combat COVID-19 and design the optimal vaccination strategies to maintain immunity in the general population.

## Figures and Tables

**Table 1:** Showing the location and function of all the targets as well as their status in COVID-19.

Target	Location (Highly Expressed)	Function	Status in COVID-19	References
TLR7	Lung, lymph nodes, gall bladder, appendix, brain, spleen, urinary bladder	Innate immune response	Low sensitivity	[39,40]
IRF5	Lung, bone marrow, duodenum, lymph node, spleen, appendix	Interferon regulation	Dysregulation in function	[41]
IRF7	Lung, kidney, bone marrow, duodenum, lymph node, spleen, appendix	Interferon regulation	Dysregulation in function	[42]
IL6	Lung, kidney, bone marrow, esophagus, appendix, urinary bladder	Proinflammatory cytokine	Involved in cytokine release syndrome	[43]
ACE2	Lung, small intestine, testis, duodenum, gall bladder, kidney	Angiotensin regulation	SARS-CoV-2 receptor	[14]
TMPRSS2	Prostate, lung, small intestine, stomach, colon	Transmembrane protein	SARS-CoV-2 S protein priming	[14]
AR	Prostate, lung, liver, testis	Nuclear receptor protein to bind androgen	Regulate the SARS-CoV-2 host genes	[44]
ESSR	Endometrium, liver, ovary, lung, thyroid	Mediate the action of estrogen	High affinity for SARS-CoV-2 S protein	[45]

## Data Availability

All datasets used in our study were publicly submitted and are available for use by researchers without the need for ethical approval.

## Supplementary Materials

The following supporting information can be downloaded at: https://www.mdpi.com/article/10.3390/v15030657/s1, Figure S1: STRING analysis showing possible gene interactions within the pathogenesis of SARS-CoV-2; Figure S2: STRING analysis showing the association of male sex hormone receptors with the SARS-CoV-2 receptor genes; Table S1: STRING analysis parameters showing the statistical values of the interactions between the target proteins; Table S2: STRING analysis parameters showing the statistical values of the interactions between the male sex hormone receptor and immune target proteins.
