# Supplementary material for: Immune Factors Drive Expression of SARS-CoV-2 Receptor Genes Amid Sexual Disparity
Source: Viruses. 2023 Feb 28;15(3):657. doi: 10.3390/v15030657 (PMC10056434; doi:10.3390/v15030657)
Supplement: Supplementary file 1 [file viruses-15-00657-s001.zip › viruses-2222856-supplementary.pdf]

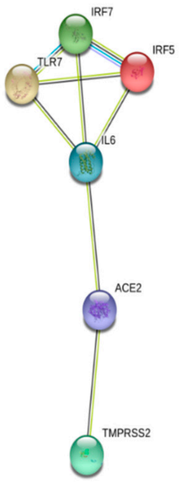

Figure S1. String analysis showing possible gene interactions within the pathogenesis of SARS-CoV-2. *TLR7* is the crucial marker in the triggering of immune response which could be activated by IRF genes. This activation could lead to the over-expression of *ACE2* and *TMPRSS2* which are necessary for the entry of SARS-CoV-2 during infection.

Table S1. String analysis parameters show the statistical values of the interactions between the target proteins. The number of edges is more than the expected number; this indicated that the proteins exhibited a higher degree of interactions with each other than what could be anticipated for a randomly selected set of proteins with a similar size and degree distribution from the genome.

**Parameters**	**Values**
the number of nodes	6
the number of edges	8
average node degree	2.67
avg. local clustering coefficient	0.75
expected number of edges	1
PPI enrichment *p*-value	3.54×10^{-6}

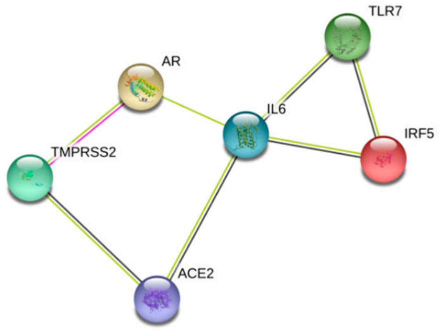

Figure S2. String analysis showing the association of male sex hormone receptors with SARS-CoV-2 receptor genes. In recent literature, AR has been suggested to regulate the expression of ACE2. The STRING analysis result showed that this AR-driven regulation of ACE2 could be regulated by cytokines.

Table S2. String analysis parameters show the statistical values of the interactions between the male sex hormone receptor and immune target proteins. The resulting number of edges was higher than expected.

Parameters	Values
the number of nodes	6
the number of edges	7
average node degree	2.33
avg. local clustering coefficient	0.361
expected number of edges	1
PPI enrichment *p*-value	0.00022
